# Efficacy and safety of haloperidol versus atypical antipsychotic medications in the treatment of delirium

**DOI:** 10.1186/1471-244X-13-240

**Published:** 2013-09-30

**Authors:** Hyung-Jun Yoon, Kyoung-Min Park, Won-Jung Choi, Soo-Hee Choi, Jin-Young Park, Jae-Jin Kim, Jeong-Ho Seok

**Affiliations:** 1 Institutional address Department of Psychiatry and Institute of Behavioral Science in Medicine, Yonsei University College of Medicine, 50 Yonsei-ro, Seoul 120-752 Seodaemun-gu, Korea; 2 Institute of Behavioral Science in Medicine, Yonsei University College of Medicine, Seoul, Korea

**Keywords:** Delirium, Haloperidol, Risperidone, Olanzapine, Quetiapine

## Abstract

**Background:**

Most previous studies on the efficacy of antipsychotic medication for the treatment of delirium have reported that there is no significant difference between typical and atypical antipsychotic medications. It is known, however, that older age might be a predictor of poor response to antipsychotics in the treatment of delirium. The objective of this study was to compare the efficacy and safety of haloperidol versus three atypical antipsychotic medications (risperidone, olanzapine, and quetiapine) for the treatment of delirium with consideration of patient age.

**Methods:**

This study was a 6-day, prospective, comparative clinical observational study of haloperidol versus atypical antipsychotic medications (risperidone, olanzapine, and quetiapine) in patients with delirium at a tertiary level hospital. The subjects were referred to the consultation-liaison psychiatric service for management of delirium and were screened before enrollment in this study. A total of 80 subjects were assigned to receive either haloperidol (N = 23), risperidone (N = 21), olanzapine (N = 18), or quetiapine (N = 18). The efficacy was evaluated using the Korean version of the Delirium Rating Scale-Revised-98 (DRS-K) and the Korean version of the Mini Mental Status Examination (K-MMSE). The safety was evaluated by the Udvalg Kliniske Undersogelser side effect rating scale.

**Results:**

There were no significant differences in mean DRS-K severity or K-MMSE scores among the four groups at baseline. In all groups, the DRS-K severity score decreased and the K-MMSE score increased significantly over the study period. However, there were no significant differences in the improvement of DRS-K or K-MMSE scores among the four groups. Similarly, cognitive and non-cognitive subscale DRS-K scores decreased regardless of the treatment group. The treatment response rate was lower in patients over 75 years old than in patients under 75 years old. Particularly, the response rate to olanzapine was poorer in the older age group. Fifteen subjects experienced a few adverse events, but there were no significant differences in adverse event profiles among the four groups.

**Conclusions:**

Haloperidol, risperidone, olanzapine, and quetiapine were equally efficacious and safe in the treatment of delirium. However, age is a factor that needs to be considered when making a choice of antipsychotic medication for the treatment of delirium.

**Trial registration:**

Clinical Research Information Service, Republic of Korea, (http://cris.nih.go.kr/cris/en/search/basic_search.jsp, Registered Trial No. KCT0000632).

## Background

Delirium is a common, complex neuropsychiatric disorder with a high prevalence among elderly hospitalized patients [1-3], postsurgical patients [4,5], and cancer patients [6-8] in advanced stages of illness. Typically, delirium shows an abrupt, rapid onset and a fluctuating course [9,10]. The core features of delirium consist of disturbances in cognitive function such as attention, memory, thought, and language [9,10]. However, its clinical presentation can be highly variable with a broad range of associated non-cognitive, behavioral symptoms that reflect the influence of distinct etiologies, medical comorbidities, or pharmacological treatments [10,11].

In hospitalized elderly patients, the prevalence of delirium ranges from 10 to 40% [3,12,13]. Delirium is also associated with major adverse outcomes such as increased mortality, functional impairment, prolonged hospitalization, and increased cost of care [14-16]. Regardless of the evident clinical significance, delirium tends to be under-diagnosed and under-treated [17]. Therefore, early identification and effective psychiatric treatment of delirium is important in the comprehensive care of elderly hospitalized patients [18].

The management of delirium includes ensuring safety with environmental or supportive interventions, identifying and treating the cause of delirium, and enhancing the patient’s functioning [19,20]. Regarding pharmacological intervention, antipsychotic medication has been considered as first-line pharmacotherapy of delirium except in the case by sedative or alcohol withdrawal [19,20]. Haloperidol, a typical antipsychotic, has continued to be the most frequently used antipsychotic drug [19-21] due to its effectiveness, relatively lesser sedative and hypotensive effects and fewer anticholinergic properties [19,21]. However, haloperidol may induce adverse side effects such as extrapyramidal symptoms (EPSs) [19,21] or prolongation of the QTc interval and fatal arrhythmia such as torsade de pointes among patients with delirium [19,21-23]. EPSs are more likely to occur in elderly and seriously medically ill patients, who are also the most susceptible to delirium [24]. In addition, it may be difficult to distinguish agitation, a common behavioral symptom of delirium, from akathisia, a frequent EPSs induced by haloperidol [25,26].

Recently, atypical antipsychotics such as risperidone, olanzapine, and quetiapine have been increasingly used to treat delirious patients due to the lower incidences of EPSs associated with these drugs [20,27-29]. Although a number of studies have been conducted to evaluate the efficacy or safety of atypical antipsychotics in the treatment of delirium, most of these reports have been in the form of case reports or open-label trials [30-32]. Only two placebo-controlled, randomized trials of atypical antipsychotics for the treatment of delirium have been reported [33,34]. Some randomized comparative trials assessing the efficacy of various antipsychotics in the treatment of delirium have been conducted in critical care units or consultation-liaison psychiatric services [26,28,32,35-39]. Five trials compared the efficacy between one atypical antipsychotic agent and haloperidol [28,35,36,38,39] and two trials compared the effectiveness of two different atypical antipsychotics [26,37]. One trial assessed the comparative efficacy among two atypical antipsychotics and haloperidol [32]. Previous trials comparing the treatment response of atypical antipsychotics based on age (<70 years old, ≥70 years old) suggested that older age might predict a poor response to the treatment of delirium [26,40]. Recently, researchers in the field of psychiatry often divide older subjects into two groups (young-old and old-old), with an age of 74 being the cutoff point [41-43]. However, no previous research has compared the response rate to various atypical antipsychotics in the treatment of delirium based on this age grouping.

Although previous trials have reported that there was no significant difference in the efficacy between haloperidol and atypical antipsychotics in the treatment of delirium [20,21,32,44], the reported data are not sufficient to form conclusions regarding the efficacy of various atypical antipsychotics compared to haloperidol. Only a few trials have considered age as a factor when comparing the response rates of atypical antipsychotics in the treatment of delirium [26,40]. To our knowledge, no previous trial has compared the efficacy and safety of haloperidol with more than two atypical antipsychotics in the treatment of delirium. For these reasons, we investigated the comparative efficacy and safety of haloperidol versus atypical antipsychotic medications in the treatment of delirium. The primary objective of this study was to compare the efficacy and safety of haloperidol versus three atypical antipsychotic medications (risperidone, olanzapine, and quetiapine) for patients with delirium. The secondary objective was to investigate whether response rate of haloperidol and three atypical antipsychotic medications differ depending on age, dividing the study cohort into two age groups, in the treatment of delirium.

## Methods

### Subject

The subjects enrolled in this study were patients presenting with a mental status change who were referred to a consultation-liaison psychiatric service at a tertiary level university hospital in Korea. To be enrolled in the study, subjects were required to meet the DSM-IV-TR [45] diagnostic criteria for delirium and to be older than 50 years. One hundred forty-six patients referred to consultation-liaison psychiatric service for a mental status change were initially included in the screening process. Among them, 130 patients who met the DSM-IV-TR [45] diagnostic criteria for delirium (77 percent) were screened for this study. Diagnosis of dementia and other psychiatric disorders was established by reviewing detailed clinical history and by obtaining information from reliable informants. Twenty-two patients with delirium were excluded. The reasons for exclusion were as follows: a diagnosis of dementia or comorbid psychiatric disorder (N = 8), a terminal illness (N = 7), a history of prolonged QTc interval (N = 3), hearing loss (N = 2), neuroleptic malignant syndrome (N = 1), and use of antipsychotic medication before referral (N = 1). Finally, 80 patients were included in this study after excluding patients (N = 11) who refused to provide informed consent.

### Assessment

The contributing cause of delirium for all participants was categorized using the Delirium Etiology Checklist (DEC) [46,47]. The DEC, which is a standardized checklist for attribution of delirium to all possible etiological causes, comprises 12 categories (drug intoxication, drug withdrawal, metabolic/endocrine disturbance, traumatic brain injury, seizures, intracranial infection, systemic infection, intracranial neoplasm, systemic neoplasm, cerebrovascular disease, organ insufficiency, other central nervous system disorders, and other systemic disease), each of which is rated on a five point scale for the degree of attribution to the episode of delirium, ranging from “ruled out/not present/not relevant” to “definite cause” [47].

The primary efficacy was evaluated by the Korean version of the Delirium Rating Scale-Revised-98 (DRS-K) [48]. The Delirium Rating Scale-Revised-98 (DRS-R-98) is an assessment tool designed for the evaluation of symptoms of delirium consisting of 16 items [49]. The DRS-R-98 is divided into two sections consisting of a 13-item severity scale and a 3-item diagnostic scale. The severity scales of DRS-R-98 include two subscales: non-cognitive (items 1–8) and cognitive (items 9–13) [50]. Each item on the severity scale is rated 0 to 3 points and each item in the diagnostic scale is rated from 0 to 2 or 3 points. The severity scale score ranges between 0 to 39 points, with a higher score indicating more severe delirium. The original validation study suggested cutoff scores for differential diagnosis from dementia or other psychiatric disorders of approximately 15 points on the severity scale [49]. Treatment response in this study was defined as ≥50% reduction from baseline in the severity scores of DRS-K in the same manner as a previous study comparing the effectiveness and safety of atypical antipsychotic medications in the treatment of delirium [26].

The secondary efficacy was evaluated by the Korean version of the Mini Mental Status Examination (K-MMSE) [51]. The Mini Mental Status Examination (MMSE) is a 30 point cognitive test for the bedside assessment of cognitive function [52]. The MMSE contains 19 items and the maximum score is 30 points (10 points for orientation, 6 points for verbal memory, 5 points for concentration and calculation, 5 points for language, 3 points for praxis, 1 point for visuospatial construction).

The safety measures involved reported adverse events and EPSs, evaluated on the basis of the Udvalg Kliniske Undersogelser (UKU) neurological side effect items (dystonia, rigidity, bradykinesia, tremor, and akathisia) [53].

All the subjects were evaluated at baseline and on the second, the fourth, and sixth days at the same time of day (PM 7:00–9:00).

### Procedure

This study was a 6-day, prospective, comparative clinical observational study of haloperidol versus three atypical antipsychotics (risperidone, olanzapine, and quetiapine) for treatment of delirium. All subjects who fulfilled the criteria were assigned either haloperidol, risperidone, olanzapine, or quetiapine group depending on the clinical and empirical judgment of the clinician. The selection of antipsychotic drug being given and dose titration were performed by one of the investigators, and all the assessments were carried out by another investigator who was blind to the antipsychotic drug being administered.

### Dose and titration

The initial starting dose was determined on the basis of age, degree of severity of delirium, and the general medical or postsurgical condition of the individual subject. The titration of dose was adjusted according to clinical judgment based on daily overall clinical impression of delirium over 6 days primarily, and was also modified depending on clinical assessments regarding the degree of improvement in delirium symptoms and the presence or absence of adverse events observed through serial assessments of DRS-K, K-MMSE, and UKU neurologic side effect items. A flexible dosing regimen (haloperidol: 0.5-10 mg, risperidone: 0.25-4 mg, olanzapine: 1–20 mg, quetiapine: 25–200 mg) was used. Because strict restriction of rescue medication in subjects with a poor general medical condition would have been ethically problematic, rescue intramuscular injections of haloperidol or lorazepam were allowed and recorded.

### Statistical analysis

The data were analyzed using the Statistical Package for Social Scientists, version 18.0 (SPSS; Chicago, IL). Group comparisons of demographic characteristics, mean baseline DRS-K and K-MMSE scores, and the mean daily chlorpromazine equivalent dose were established using the chi-square test or Fisher’s exact test for categorized variables and one-way analysis of variance (ANOVA) for continuous variables. In this longitudinal study, because some data were missing due to dropout or other reasons such as discharge, a linear mixed model [54,55] was applied to compare changes in DRS-K and K-MMSE scores during treatment within each group and among the four groups. This model takes into account all available data, allows for missing value, and estimates fixed effects while adjusting for correlation due to repeated measurements on each subject [54-56]. Medication group, visit day, and day-by-group interaction were included as fixed effects. The group difference in treatment response and side effect profile were analyzed by the chi-square test or Fisher’s exact test. In order to examine differences in treatment response depending on age, each patient’s age was converted to a dichotomous variable with two levels (young-old: <75 years old, old-old: ≥75 years old) [41-43]. All statistical analyses were two-tailed, with a significance level of probability set at 0.05.

### Consent and approval

The study design was approved by the Institutional Review Board and the ethics committees at Gangnam Severance Hospital, Yonsei University College of Medicine, Seoul, Korea. Written informed consent was obtained either from the subjects’ primary caregivers or the legal representatives of the subjects prior to enrollment. Although it is the best way to seek consent from the subjects themselves in the clinical research, patients in the episode of delirium were unable to communicate meaningfully in most cases actually. Thus, we were obliged to seek consent from the primary caregivers or the legal representatives of the subjects. Prior to screening, the objective of this study and the pharmacological treatments available were explained to them. The primary caregivers or the legal representatives of subjects had the right to withdraw consent at any time during this study. Other ethical safeguards were also maintained during the study.

The other ethical safeguards maintained for the study is as follows: The etiological condition identified as cause of delirium was corrected and treated appropriately. Treatment for the primary medical or surgical condition of subjects was continued during study period in addition to use of the trial antipsychotic medications. Any medication which could cause or aggravate delirium was discontinued promptly since screening process of study began. Any medication that was not essential for the treatment of underlying condition was used minimally or stopped during the study period. Personal information of the subjects was stored at the separate disc space with password.

## Results

### Demographic characteristics and causes of delirium

A total of 80 patients consisting of 36 men (45%) and 44 women (55%) were enrolled in this study. The mean age and years of education of the subjects were 71.8 ± 11.5 years and 7.5 ± 5.6 years, respectively. There were no significant differences in sex, age and years of education between the four groups. In regard to contributing etiological causes of delirium, the mean number of all possible causes per patient was about two (1.9 ± 0.6). Most patients had two (N = 52, 87.5%) or three (N = 10, 12.5%) contributing causes of delirium, and eighteen patients (22.5%) had one definite cause associated with delirium. Significant difference was not observed on number of contributing causes between the four groups. The most common definite etiology of delirium in the study sample was metabolic-endocrine abnormality (N = 23). This was followed by organ insufficiency (N = 16), systemic infection (N = 14), systemic neoplasm (N = 12), cerebrovascular cause (N = 9), and others (N = 6). A comparison of the frequency of each definite etiology of delirium among the four groups revealed that the differences among the four groups were not statistically significant (Table 1).

**Table 1:** Group comparisons of demographic characteristics, causes of delirium, medication, and number of subjects assessed

**Characteristic**		**Haloperidol**	**Risperidone**	**Olanzapine**	**Quetiapine**	**Total**	**Sig.**
		**N = 23**	**N = 21**	**N = 18**	**N = 18**	**N = 80**	
Age, year		74.0 ± 9.9	70.1 ± 9.5	69.5 ± 15.9	73.3 ± 10.7	71.8 ± 11.5	0.522
Education, year		5.8 ± 4.5	8.7 ± 6.9	8.5 ± 6.4	7.3 ± 3.9	7.5 ± 5.6	0.327
Gender, male		12(52.2)	8(38.1)	8(44.4)	8(44.4)	36(45)	0.828
Number of contributing causes of delirium		1.8 ± 0.5	1.9 ± 0.7	1.9 ± 0.4	2.0 ± 0.8	1.9 ± 0.6	0.783
Definite cause of delirium							
Metabolic/endocrine		6(26.0)	8(38.0)	4(22.2)	5(27.7)	23(28.7)	0.759
Systemic infection		4(17.3)	3(14.2)	3(16.6)	4(22.2)	14(17.5)	0.957
Systemic neoplasm		6(26.0)	1(4.7)	3(16.6)	2(11.1)	12(15.0)	0.260
Cerebrovascular		3(13.0)	3(14.2)	0(0.0)	3(16.6)	9(11.2)	0.328
Organ insufficiency		3(13.0)	3(14.2)	8(44.4)	2(11.1)	16(20.0)	0.059
Others		1(4.3)	3(14.2)	0(0.0)	2(11.1)	6(7.5)	0.354
Dose, mg/day		1.2 ± 0.4	1.1 ± 0.3	2.9 ± 1.0	47.9 ± 17.1		
Chlorpromazine equivalent dose, mg/day		60.0 ± 21.4	56.3 ± 16.8	59.8 ± 20.5	63.9 ± 22.8	59.8 ± 20.4	0.192
Duration of medication, day		4.7 ± 1.6	5.1 ± 1.3	5.3 ± 1.1	4.8 ± 1.7	4.9 ± 1.5	0.655
Number of subjects assessed,	Baseline	23(100.0)	21(100.0)	18(100.0)	18(100.0)	80(100.0)	
	Day 2	18(78.2)	21(100.0)	18(100.0)	15(83.3)	72(90.0)	
	Day 4	16(69.5)	18(85.7)	15(83.3)	12(66.6)	61(76.2)	
	Day 6	14(60.8)	14(66.6)	13(72.2)	12(66.6)	53(66.2)	

Data were presented as mean ± SD or N(%).

### Treatment group and clinical course of delirium

All subjects (N = 80) were assigned to receive either haloperidol (N = 23), risperidone (N = 21), olanzapine (N = 18), or quetiapine (N = 18) according to the clinical judgment of the investigator at the baseline assessment. Of the 80 subjects enrolled, 53 patients (66.2%) completed this trial. The reasons for drop out included loss of follow up due to discharge from hospital (N = 18), transfer to the intensive care unit (N = 6), and withdrawal of consent (N = 3). In the haloperidol group, nine of 23 patients dropped out during the study. Five subjects were discharged from the hospital, two subjects were transferred to the intensive care unit, and two subjects withdrew consent. In the risperidone group, seven of 21 subjects could not be evaluated after the fourth day because they were discharged from the hospital (N = 5) or transferred to the intensive care unit (N = 2). In the olanzapine group, five of 18 subjects did not complete the trial because of discharge from the hospital (N = 4) or transfer to the intensive care unit (N = 1). In the quetiapine group, six of 18 subjects dropped out due to discharge from the hospital (N = 4), transfer to the intensive care unit (N = 1), or withdrawal of consent (N = 1). The difference in the dropout rate was not significant among the four groups (p = 0.899). Excluding cases of dropout, the numbers of subjects who could not be evaluated at least once after the baseline assessment due to loss to follow-up or worsening medical condition were six in the haloperidol group, one in the risperidone group, two in the olanzapine group, and five in the quetiapine group.

The difference in the chlorpromazine equivalent dose between the four groups was not significant (p = 0.192).

Rescue intramuscular injection of haloperidol was used 13 times in seven subjects in the haloperidol group, 12 times in nine subjects in the risperidone group, eight times in four subjects in the olanzapine group, and 15 times in nine subjects in the quetiapine group. The mean doses of rescue intramuscular haloperidol were similar among the four groups (haloperidol: 1.4 ± 2.3 mg, risperidone: 1.4 ± 1.8 mg, olanzapine: 1.1 ± 2.6 mg, quetiapine: 2.3 ± 2.6 mg, p = 0.419). Rescue intramuscular lorazepam injection was used once in one subject in the haloperidol group, twice in two subjects in the risperidone group, and four times in two subjects in the olanzapine group.

The mean duration of medication among all subjects was 4.9 ± 1.5 days. The mean duration of medication was not significantly different among the four groups (haloperidol: 4.7 ± 1.6 days, risperidone: 5.1 ± 1.3 days, olanzapine: 5.3 ± 1.1 days, quetiapine: 4.8 ± 1.7 days, p = 0.655).

### Efficacy analysis

**Figure 1:**
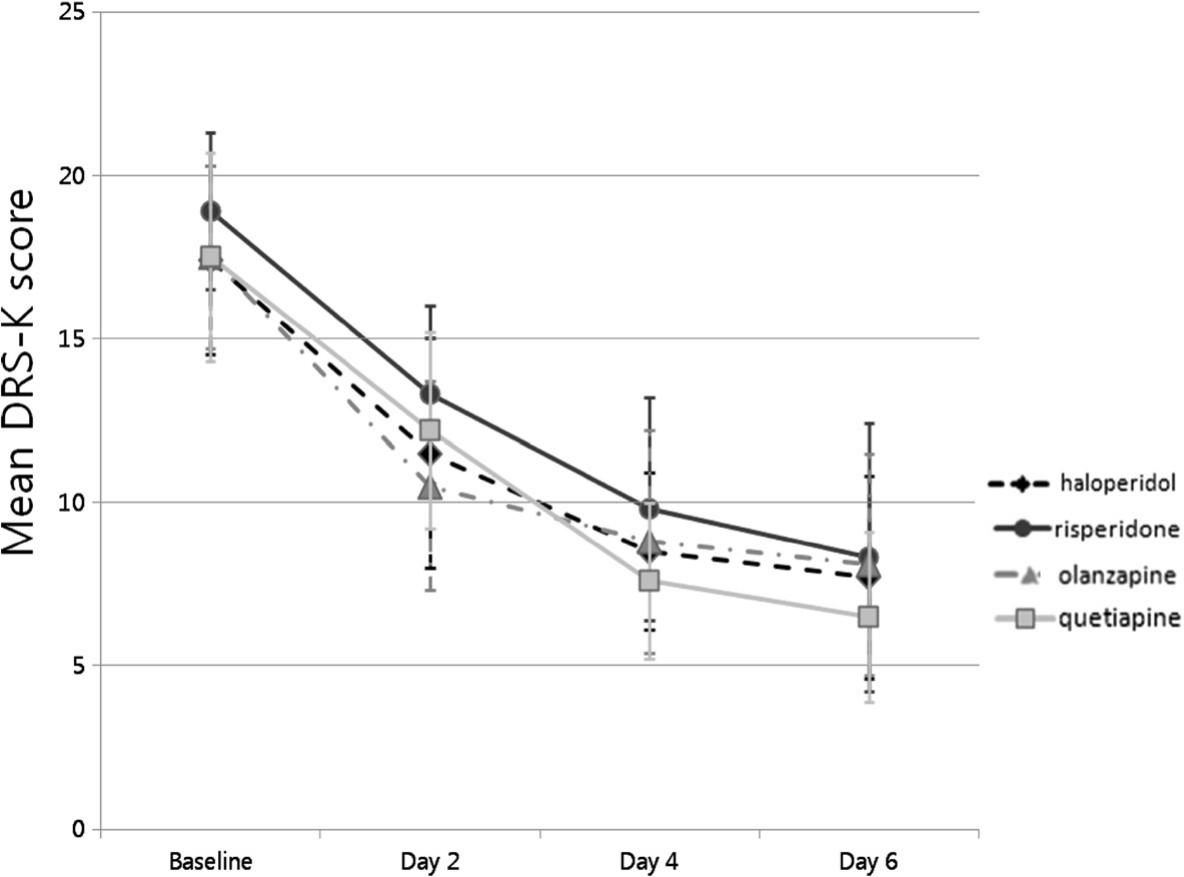
**Serial changes in DRS-K severity scores in the four antipsychotic groups.** Mean changes in DRS-K severity scores with 95% confidence intervals. In all antipsychotic groups, the mean DRS-K severity score decreased significantly over the study period (all p-values analyzed by linear mixed model statistics < 0.0001). However, there were no significant differences in the degree of reduction in mean DRS-K severity score with time among the four groups (p-values analyzed by linear mixed model statistics = 0.779).

**Figure 2:**
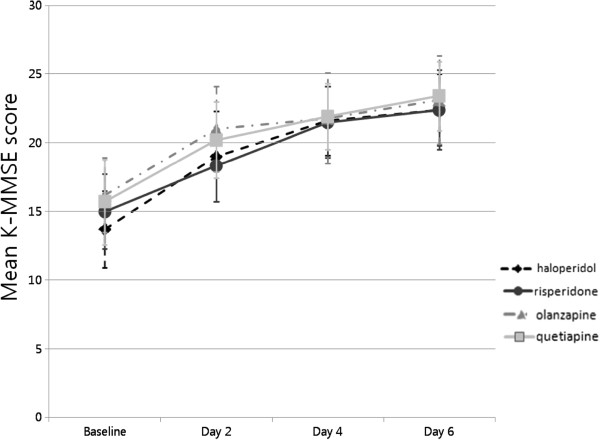
**Serial changes in K-MMSE scores in the four antipsychotic groups.** Mean changes in K-MMSE scores with 95% confidence intervals. In all antipsychotic groups, the mean K-MMSE score increased significantly over the study period (all p-values analyzed by linear mixed model statistics < 0.0001). However, there were no significant differences in the degree of improvement in mean K-MMSE score with time among the four groups (p-values analyzed by linear mixed model statistics = 0.630).

In regards to both the primary and secondary efficacy measures of this study, the within-group effect was statistically significant in all groups. A significant serial decrease in the mean DRS-K severity score (Figure 1) and increase in the mean K-MMSE score (Figure 2) was observed in all groups during the study period. The day-by-group interaction effect and between-group effect was not significant in any efficacy measures (Table 2).

**Table 2:** Group comparisons of serial changes in DRS-K and K-MMSE scores

**Efficacy measures**		**Haloperidol**	**Risperidone**	**Olanzapine**	**Quetiapine**	**Total**	**Sig.**
DRS-K							
Severity score	Baseline	17.4 ± 6.7	18.9 ± 5.2	17.5 ± 5.7	17.5 ± 6.4	17.8 ± 6.0	0.779^**†**^
	Day 2	11.5 ± 7.1	13.3 ± 5.8	10.5 ± 6.6	12.2 ± 5.4	11.93 ± 6.2	
	Day 4	8.5 ± 4.6	9.8 ± 6.7	8.8 ± 6.0	7.6 ± 3.7	8.80 ± 5.4	
	Day 6	7.7 ± 5.4	8.3 ± 7.1	8.1 ± 5.5	6.5 ± 4.0	7.75 ± 5.5	
Cognitive subscale score	Baseline	7.8 ± 3.8	8.7 ± 3.4	7.7 ± 3.6	8.1 ± 3.2	8.14 ± 3.5	0.718^**†**^
	Day 2	5.7 ± 3.9	6.5 ± 3.4	5.0 ± 3.0	5.6 ± 2.6	5.76 ± 3.3	
	Day 4	4.3 ± 2.4	4.8 ± 3.6	4.2 ± 3.2	4.0 ± 2.5	4.43 ± 3.0	
	Day 6	4.0 ± 2.9	4.1 ± 4.0	4.2 ± 2.7	3.2 ± 2.5	3.94 ± 3.1	
non-cognitive subscale score	Baseline	9.5 ± 3.5	10.1 ± 3.0	9.7 ± 3.3	9.4 ± 4.2	9.73 ± 3.4	0.918^**†**^
	Day 2	5.7 ± 3.5	6.5 ± 2.9	5.5 ± 3.8	6.6 ± 3.7	6.11 ± 3.4	
	Day 4	4.1 ± 2.6	4.9 ± 3.8	4.6 ± 3.5	3.6 ± 2.2	4.41 ± 3.1	
	Day 6	3.7 ± 2.8	4.2 ± 3.6	3.9 ± 3.5	3.3 ± 2.0	3.81 ± 3.0	
K-MMSE score							
	Baseline	13.7 ± 6.5	15.0 ± 5.8	16.2 ± 5.4	15.7 ± 6.3	15.1 ± 6.0	0.630^**†**^
	Day 2	19.0 ± 6.7	18.3 ± 5.7	21.0 ± 6.2	20.2 ± 4.9	19.5 ± 5.9	
	Day 4	21.3 ± 4.7	21.5 ± 5.3	21.8 ± 5.8	21.9 ± 3.7	21.6 ± 4.9	
	Day 6	22.4 ± 4.4	22.4 ± 5.0	23.1 ± 5.3	23.4 ± 3.7	22.8 ± 4.6	

Data were presented as mean ± SD.

†: p-values analyzed among groups by linear mixed model statistics.

Each day-by-group interaction was not significant in all efficacy measures.

(all p-values analyzed by linear mixed model statistics >0.05).

In all medication groups, the mean score of DRS-K severity, cognitive and non-cognitive subscale tended to decrease significantly over study period.

(all p-values analyzed by linear mixed model statistics < 0.0001).

In all medication groups, the mean score of K-MMSE tended to increase significantly over the study period. (all p-values analyzed by linear mixed model statistics < 0.0001).

The cognitive and non-cognitive subscale scores of the DRS-K decreased significantly over the study period in all groups (p < 0.001). However, the rate of reduction of either subscale score did not differ significantly among the four groups during the study period (p = 0.718, p = 0.918).

**Figure 3:**
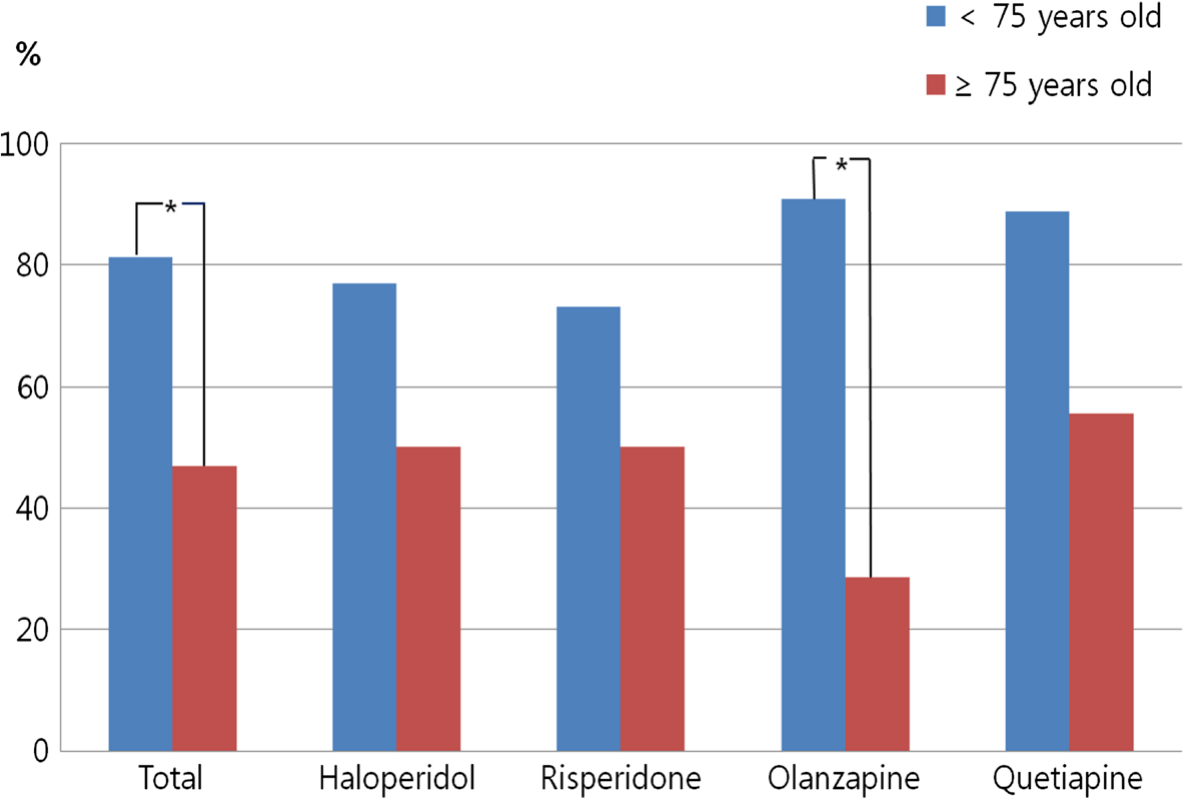
**Treatment response rate between young-old and old-old groups in the four antipsychotic groups.** * p < 0.05 by Chi-square test or Fisher’s exact test. Treatment response was defined as a ≥50% reduction from the baseline DRS-K score.

In terms of treatment response, there was no significant difference in the response rate among the four groups (haloperidol: 15/23, 65.2%, risperidone: 14/21, 66.6%, olanzapine: 12/18, 66.6%, and quetiapine: 13/18, 72.2%, p = 0.969). When response rate was compared according to demographic characteristics, no significant difference was noted according to sex (p = 0.886). Overall, the response rate was significantly lower in subjects over 75 years old (15/32, 46.8%) compared to those under 75 years old (39/48, 81.2%, p = 0.001) (Figure 3). Of note, the response rate to olanzapine was much lower in subjects over 75 years old (2/7, 28.5%) compared to those under 75 years old (10/11, 90.9%, p = 0.013), while the response rates of the other three groups did not differ significantly between the two age groups (p > 0.05) (Figure 3).

The difference in the mean baseline K-MMSE score among the four groups was not significant (p = 0.565). In contrast to the DRS-K severity score, the mean K-MMSE score increased serially from the baseline assessment in all groups (all p < 0.001). However, the rate of improvement in the K-MMSE score did not differ significantly among the four groups (p = 0.630).

### Safety analysis

Overall, all subjects tolerated the four antipsychotics well. Fifteen (18.8%) of the total subjects experienced some adverse events. Exacerbation of sedation or sleepiness was reported in four subjects in both the haloperidol and olanzapine groups, three subjects in the risperidone group, and two subjects in the quetiapine group. Rigidity was reported in two subjects in the haloperidol group, and one subject in each of the other three groups. Bradykinesia was reported in one subject in each of the haloperidol, risperidone, and olanzapine groups. Tremors were reported in three subjects in the haloperidol group, two subjects in the risperidone group, and one subject in each of the olanzapine and quetiapine groups. Akathisia was only reported in one subject in the haloperidol group. All extrapyramidal side effects were tolerable and mild in severity. When the number of subjects experiencing side effects was compared among the four groups, the difference was not statistically significant (Table 3).

**Table 3:** Group comparisons of frequency of UKU side effect rating scale items

**Side effects**	**Haloperidol**	**Risperidone**	**Olanzapine**	**Quetiapine**	**Total**	**Sig.**
Sedation/Sleepiness	4(17.3)	3(14.2)	4(22.2)	2(11.1)	13(16.2)	0.838
Dystonia	0(0.0)	0(0.0)	0(0.0)	0(0.0)	0(0.0)	-
Rigidity	2(8.7)	1(4.7)	1(5.5)	1(5.5)	5(6.2)	1.000
Bradykinesia	1(4.3)	1(4.7)	1(5.5)	0(0)	3(3.7)	1.000
Tremor	3(13.0)	2(9.5)	1(5.5)	1(5.5)	7(8.7)	0.869
Akathisia	1(4.3)	0(0)	0(0)	0(0)	1(1.2)	1.000
Total number of subjects reporting side effects	5(21.7)	4(19.0)	4(22.2)	2(11.1)	15(18.7)	0.857

Data were presented as N(%).

## Discussion

Recently, a number of researchers have reported that atypical antipsychotics may be as effective as haloperidol in treating delirium [20,21,28,32,35,36,38,39]. Risperidone, olanzapine, and quetiapine have been increasingly used for pharmacologic intervention of delirium [20,26-29]. Previous researches have shown that the efficacy of risperidone and olanzapine is not different from that of haloperidol in the treatment of delirium [28,32,35,36,44,57-59]. Quetiapine has been reported to be as efficacious as haloperidol [58] and to reduce the severity of the symptoms of delirium more rapidly than placebo [33]. To date, most randomized comparative trials of atypical antipsychotics in the treatment of delirium have compared the efficacy of one atypical antipsychotic agent and haloperidol [28,35,36,38,39] or two different atypical antipsychotics [26,37]. Only one randomized comparative study has compared the efficacy of two different atypical antipsychotics and haloperidol [32]. To our knowledge, this study is the first trial to compare the efficacy and safety of haloperidol versus three atypical antipsychotics. In the present study, haloperidol, risperidone, olanzapine and quetiapine were equally effective in improving the symptoms of delirium. There was no significant difference in the rate of reduction of DRS-K severity score and improvement of K-MMSE score with time among the four groups. Recently, one comparative efficacy study of haloperidol, risperidone, and olanzapine showed that risperidone and olanzapine were as efficacious as haloperidol in treating delirium [32]. Our result supports the findings of previous researches with regard to the comparative efficacy of haloperidol versus three atypical antipsychotics in managing symptoms of delirium [28,32,35,36,44,57-59]. The mean daily doses of risperidone, olanzapine, and quetiapine were not largely different from those of previous studies [26,32,35,36,57,60-62]. This finding also suggests that a relatively low dose of atypical antipsychotics may be effective in managing the symptoms of delirium [26,28,32,36].

There have been no previous studies to have assessed the difference in the efficacy of atypical antipsychotics in terms of the cognitive and non-cognitive subscale scores of the DRS-R-98, with the exception of one placebo, controlled trial [33]. The group difference was not significant over this study period in terms of the rate of reduction of the cognitive and non-cognitive subscale scores of the DRS-K. In fact, improvement of cognitive and non-cognitive symptoms of delirium could occur naturally [33], due to nonspecific environmental care [63] or to treating the underlying etiologies of delirium [21]. Nevertheless, our findings might be meaningful in that the effectiveness of haloperidol versus three atypical antipsychotics was compared in the two different symptom domains of delirium at the same time in a real clinical setting.

The demographic characteristics of the subjects enrolled in this study were not significantly different from those of subjects in previous studies [32,36,37]. In regards to the causes of delirium, most subjects had more than one contributing cause of delirium. The most common definite cause of delirium was metabolic or endocrine disturbance, a finding which was similar to that of previous studies of patients with delirium referred to a consultation-liaison psychiatric service [32,47].

In the present study, the response rate to olanzapine was poor in subjects over 75 years old compared to those under 75 years old. However, the response rate to the other three antipsychotics was not significantly different between age groups. A previous study reported that old age was associated with poorer response to olanzapine in hospitalized cancer patients with delirium [40], while another study reported that the response rate of olanzapine was similar depending on age, but that the response rate to risperidone was much lower in an older age group [26]. The major neurotransmitter hypothesized to be involved in delirium is acetylcholine, and several studies have reported that a variety of delirium-inducing factors are associated with decreased acetylcholine activity in the brain [64]. Actually, many medications with anticholinergic side effects can induce or aggravate delirium [21]. In regards to the pharmacological profile, olanzapine is known to have a significant affinity for muscarinic receptors and to induce relatively more anticholinergic adverse effects than the other three antipsychotics [65,66]. Age-related differences in susceptibility to anticholinergic adverse effects might have affected the response rate in the olanzapine group. Another possibility for the reduced effectiveness of olanzapine in the older age group is that some of the patients may have had undiagnosed dementia. In addition, this might be related to differences in the general underlying medical condition, as the frequency of organ insufficiency was relatively higher in the olanzapine group than in the other three groups. These results suggest that advanced age is not only a risk factor [21,64,67] for delirium, but also may be a predictor of poor response to delirium treatment with atypical antipsychotics. Therefore, further investigation of the impact of age on treatment response is required.

There were no significant group differences in the number of subjects experiencing adverse events or in the type of adverse events. Although previous review articles have suggested that atypical antipsychotics are safe, with a lower rate of adverse events compared to haloperidol in the treatment of delirium [20,21], a Cochrane review reported that haloperidol at a low dosage (<3.5 mg/day) was safe, with a similar frequency of adverse events compared to atypical antipsychotics [44]. In this study, the mean daily dose of haloperidol was relatively low (1.2 ± 0.4 mg/day) and the total duration of medication was relatively short. Thus, the results of the present study suggest that a low dose of haloperidol is safe and does not show a greater frequency of EPSs [44] compared to atypical antipsychotics over a relatively short period of treatment.

This study has several limitations: ⓐ Our study did not include a placebo control group. The absence of comparative placebo control group with active treatment groups limited the interpretation of our findings; ⓑ The use of empirical judgment of clinician to assign antipsychotic medications without randomization might be important source of bias and limit our findings. However, we found no significant difference in demographic and clinical characteristics among four groups; ⓒ Heterogeneity of study population including medical and surgical patients could be a major limiting factor in the interpretation of our main findings even though there was no significant difference in clinical characteristics among four groups; ⓓ Since the consultation-liaison psychiatric service might recruit a skewed patient cohort most likely to have active psychiatric symptoms, this should be acknowledged as a source of bias; ⓔ Since this was a pilot study, we did not consider sample size as a requirement for carrying out the proposed objectives and the small subject numbers in each group might limit the strength of the conclusions from our work. Thus, further studies with a larger number of subjects are needed to test our findings; ⓕ Although we made efforts to exclude patients with dementia by reviewing detailed clinical history and by obtaining information from reliable informants, dementia was not evaluated by a standardized screening instrument. Given that the number of subjects with dementia was relative low (N = 8) in screening process, subjects with dementia might be misclassified; ⓖ Although the rater was blind to which study drug was being administered, as the rater knew that all subjects were receiving active treatment, the ratings could have been affected; ⓗ Confounding factors associated with rescue medication could not be rigidly controlled due to ethical considerations. The permission to use rescue medication might not have seriously affected the results of this study because the total mean dose of intramuscular rescue injection of haloperidol was not significantly different among the four groups. If the dosing titration of the study drugs had been escalated more rapidly, the need for rescue medication could have been decreased; ⓘ The dropout rate was relatively high, and the missing data caused by dropouts could affect the result of this study. In order to overcome this limitation, we used a linear mixed model, in which all available data can be included and missing data can be appropriately addressed [54,55]. By using the average area under each subject’s rating scale trajectory, we could compare treatment groups across whole study period; ⓙ The safety analysis was focused only on extrapyramidal side effects and could not evaluate other potential adverse events, such as QTc prolongation or arrhythmia associated with use of antipsychotic drugs [19,21-23]. Overall, six patients were transferred to the intensive care unit due to aggravation of an underlying medical or postsurgical condition during the study. Given that potential adverse effects of antipsychotic drugs, which had not been captured by data, might contribute to aggravation of underlying medical conditions, transfer to the intensive care unit during the study needs to be considered as a potential adverse effect of antipsychotic medications; ⓚ Changes in the various medical or surgical conditions of the study subjects might have affected the symptom severity of delirium, regardless of the use of antipsychotics; ⓛ Finally, as the symptoms of delirium can fluctuate and improve irrespective of the treatment given [33], the findings of our study must be understood in the background of these limitations. Regardless of these limitations, the results of the present study could provide important clinical information regarding the usefulness of commonly prescribed antipsychotics in the treatment of delirium with various underlying etiologies in a tertiary hospital setting.

## Conclusions

In conclusion, the atypical antipsychotics risperidone, olanzapine, and quetiapine and low dose haloperidol were equally effective and safe in the treatment of delirium. The treatment response rate for the only olanzapine group was significantly lower in subjects over 75 years old than in subjects under 75 years old. The factor of age needs to be considered in the choice of antipsychotic medication for the treatment of delirium. Further prospective randomized placebo controlled trials of a larger patient group with delirium should be carried out to test the generalizability of our findings.

## Abbreviations

EPSs: Extrapyramidal symptoms; DSM-IV-TR: Diagnostic and statistical manual of mental disorders-iv-text revision; DEC: Delirium etiology checklist; DRS-K: Korean version of the delirium rating scale-revised-98; DRS-R-98: Delirium rating scale-revised-98; K-MMSE: Korean version of the mini mental status examination; MMSE: Mini mental status examination; UKU: Udvalg Kliniske Undersogelser; SPSS: Statistical package for social scientists; ANOVA: One-way analysis of variance.

## Competing interests

The result of this study was submitted as a research poster in the 16th international congress of international psychogetriatric association in Seoul.

## Authors’ contributions

All authors participated in the design of the trial and read and approved the final manuscript.

## Pre-publication history

The pre-publication history for this paper can be accessed here:

http://www.biomedcentral.com/1471-244X/13/240/prepub
